# Increased B and T Cell Responses in *M*. *bovis* Bacille Calmette-Guérin Vaccinated Pigs Co-Immunized with Plasmid DNA Encoding a Prototype Tuberculosis Antigen

**DOI:** 10.1371/journal.pone.0132288

**Published:** 2015-07-14

**Authors:** Nicolas Bruffaerts, Lasse E. Pedersen, Gaëlle Vandermeulen, Véronique Préat, Norbert Stockhofe-Zurwieden, Kris Huygen, Marta Romano

**Affiliations:** 1 Service Immunology, Scientific Institute for Public Health (WIV-ISP Site Ukkel), Brussels, Belgium; 2 Section for Immunology and Vaccinology, Technical University of Denmark, Bulowsvej Frederiksberg C, Denmark; 3 Louvain Drug Research Institute, Pharmaceutics and Drug Delivery, Université Catholique de Louvain, Brussels, Belgium; 4 Division Infection Biology Central Veterinary Institute, Part of Wageningen U(niversity)&R(esearch); Lelystad, The Netherlands; Public Health England, UNITED KINGDOM

## Abstract

The only tuberculosis vaccine currently available, bacille Calmette-Guérin (BCG) is a poor inducer of CD8^+^ T cells, which are particularly important for the control of latent tuberculosis and protection against reactivation. As the induction of strong CD8^+^ T cell responses is a hallmark of DNA vaccines, a combination of BCG with plasmid DNA encoding a prototype TB antigen (Ag85A) was tested. As an alternative animal model, pigs were primed with BCG mixed with empty vector or codon-optimized pAg85A by the intradermal route and boosted with plasmid delivered by intramuscular electroporation. Control pigs received unformulated BCG. The BCG-pAg85A combination stimulated robust and sustained Ag85A specific antibody, lymphoproliferative, IL-6, IL-10 and IFN-γ responses. IgG1/IgG2 antibody isotype ratio reflected the Th1 helper type biased response. T lymphocyte responses against purified protein derivative of tuberculin (PPD) were induced in all (BCG) vaccinated animals, but responses were much stronger in BCG-pAg85A vaccinated pigs. Finally, Ag85A-specific IFN-γ producing CD8^+^ T cells were detected by intracellular cytokine staining and a synthetic peptide, spanning Ag85A_131-150_ and encompassing two regions with strong predicted SLA-1*0401/SLA-1*0801 binding affinity, was promiscuously recognized by 6/6 animals vaccinated with the BCG-pAg85A combination. Our study provides a proof of concept in a large mammalian species, for a new Th1 and CD8^+^ targeting tuberculosis vaccine, based on BCG-plasmid DNA co-administration.

## Introduction

The only vaccine currently available to prevent tuberculosis (TB) is the live, attenuated *M*. *bovis* Bacille Calmette-Guérin (BCG) vaccine. BCG vaccination protects children against TB meningitis and against disseminated, miliary disease, but confers a variable protection (ranging from 0% to 80%) against pulmonary TB [1,2]. Clearly, there is a need for a more efficacious TB vaccine for both prophylactic and post-exposure use [3]. Although a Th1 type CD4^+^ mediated immune response is essential for protection against tuberculosis (as indicated by the increased risk for TB in HIV-co-infected individuals), CD8^+^ T cells are also important, especially for the control of a latent tuberculosis infection and prevention of reactivation [4,5]. BCG vaccination is only a weak inducer of CD8^+^ T cells as compared to tuberculosis infection. Indeed, BCG carries numerous genes that act to dampen CD8^+^ T cell responses [6] and a 200-fold higher dose of BCG is needed to induce CD8^+^ responses comparable in magnitude to those induced with *M*. *tuberculosis* [7]. The induction of robust CD8^+^ responses requires the use of the endogenous antigen presentation pathway, as it is triggered by live pathogens (e.g. *M*. *tuberculosis*) or live attenuated viral vaccines (e.g. recombinant adenoviruses). By virtue of their capacity to use this endogenous antigen presentation pathway, plasmid DNA vaccines can elicit robust MHC class I restricted CD8^+^ responses (besides inducing also strong MHC class II restricted Th1 type CD4^+^ responses). This makes them particularly attractive as vaccine delivery systems against intracellular pathogens, such as mycobacteria [8] [9][10]. It has previously been shown in mice and cattle that priming with DNA vaccines encoding TB antigens *prior* to BCG, can improve the potency of the BCG vaccine [11] [12,13] [14] [15]. However, priming with DNA and boosting with BCG is an unrealistic vaccine regimen in humans as BCG is routinely given to neonates. As an alternative, we have examined a *simultaneous* co-administration of BCG with plasmid DNA encoding an *M*. *tuberculosis* antigen. As a prototype mycobacterial antigen for this study, we selected the mycolyl-transferase Ag85A (Rv3804c) [16]. Together with Ag85B (Rv1886c), these two proteins are among the most studied vaccine antigens of *M*. *tuberculosis*, present abundantly in mycobacterial culture filtrate. The Ag85A component of *M*. *tuberculosis* can induce strong T cell proliferation and IFN-γ production in most healthy individuals infected with *M*. *tuberculosis* / *M*. *leprae* and in BCG vaccinated mice, but not in tuberculosis or lepromatous leprosy patients [17]. In mice and guinea pigs, members of the Ag85 family were demonstrated to be promising candidates for future TB vaccines [18,19] and more than half of the vaccine candidates that successfully boosted BCG in preclinical studies contain these antigens [20]. Nevertheless, two phase 2b clinical trials of MVA85A failed to increase protection conferred by BCG, despite remarkably persistent vaccine-induced Ag85A-specific CD4^+^ T cell responses in healthy, HIV-uninfected adults, adolescents, children and infants, up to 6 years after booster vaccination [21–23]. The choice of antigen, tissue location, epidemiological or clinical factors and the high rate of *M*.*tuberculosis* transmission in the two trial populations all may underlie this lack of vaccine-induced protection. However, it is also possible that the rationale for boosting a *waning* immune response is flawed and that BCG induces an *wrong/ incomplete* (i.e. exclusively CD4^+^ focused) immune response which can no longer be redirected/completed by MVA85A boosting [24].

For this study, we choose domestic pigs, which are closely related to humans in terms of anatomy, genetics and physiology and which resemble humans for > 80% of immune parameters (vs. < 10% for mice) [25]. Pigs are highly relevant for skin studies, as epidermal thickness and dermal: epidermal thickness ratio is comparable to human, which has obvious relevance for a vaccine routinely administered by the intradermal route. When injected with naked DNA, pig skin transiently expresses the injected gene at high levels in the epidermis and produces biologically active protein (eg. cytokines)[26]. Also quantitative expression of the plasmid encoded protein is significantly higher in pig (and human) skin than in mouse skin, which appears to take-up and express the injected plasmid DNA at multiple sites besides the epidermis [27]. Furthermore, it was previously reported that mycobacteria-specific T cell responses can be induced by BCG vaccination in 4 week old piglets [28]. Thus, both γδ and CD4^+^ T cell mediated IFN-γ production could be detected after stimulation with culture filtrate protein, as well as innate and acquired antigen-specific γδ and CD8^+^ T cell mediated cytolytic activity against autologous BCG infected monocytes [29].

In this study, three groups of six animals each, were primed intradermally with *M*. *bovis* BCG alone (group 1), BCG mixed with empty control vector (group 2) or BCG mixed with codon-optimized V1J.ns-tPA-Ag85A vector (group 3). Groups 2 and 3 received two additional intramuscular booster vaccinations of plasmid DNA coupled to *in vivo* electroporation. Mycobacteria-specific humoral and cellular immune responses were analyzed prior to and at 4 time points after vaccination, over a period of 118 days.

## Materials and Methods

### Animals

Eighteen pigs (*Sus scrofa domestica*, line TOPIGS20*)* of ten weeks of age at day 0, were supplied by a conventional pig farm with a high health status (Geert van Beek B.v., Runderweg, Lelystad, NL); the herd has an SPF status, which is defined by the breeding organization (TOPIGS, NL) and is free of common swine pathogens. The study was approved by the Animal Ethical Committee of WUR (Study Approval nr.: 2012113).

### Plasmids

tPA-flagged antigen 85A DNA sequence [18] was codon optimized for pig, upgrading the CAI to 0.81, and was optimized considering GC content reduction, stem-loop structures break, and negative cis-acting sites (GenScript, Piscataway, USA). Next, the gene was cloned into pV1J.ns vector [30] and amplified in *E*. *coli* DH5-α, before purification with PureLink HiPure Gigaprep kit (Life Technologies, Carlsbad, USA). Empty pV1J.ns-tPA vector was used as control.

### Immunization

Three groups of six animals each were allocated to the different treatment groups by stratified randomization based on animal weight (Table 1). All pigs were housed in one animal room and each treatment groups was kept in one pen (CVI, wing 043, Edelhertweg 15, NL-8219 PH Lelystad). No blinding to vaccination or challenge period was applied.

**Table 1:** 

Group number	1	2	3
**Initial weight**	18.3 ± 2.2 kg	19.0 ± 3.1 kg	19.6 ± 2.7 kg
**d0**	BCG (i.d.)	BCG mixed with 500 μg empty vector (i.d.)	BCG mixed with 500 μg pAg85A (i.d.)
**d21, d42**	-	1000 μg empty vector (i.m. with electroporation)	1000 μg pAg85A (i.m. with electroporation)

On day 0, BCG Danish 1331 (Staten Serum Institute SSI) 2x10^6^ CFU (i.e. the equivalent of ten human doses, taken into account the initial piglet weight) per animal was delivered intradermally about 10 cm below the right *tuber ischiadicum* in two adjacent areas of the skin; per injection site 125 μl was administered slowly. For groups 2 and 3, BCG was dissolved in empty vector or codon optimized pAg85A respectively (prepared at a concentration of 2 mg/mL in PBS and stored at -20°C until use) to achieve a final dose of 500 μg/250 μl. Due to financial constraints, a fourth group of pigs only vaccinated with pAg85A DNA could not be included.

On day 21 and day 42, animals of groups 2 and 3 were anaesthetized with intravenously administered propofol (10mg/mL, Propovet, Abbott, GB) and the plasmid (diluted in PBS) was administered intramuscularly in the right thigh at about the same location as the intradermal BCG injection in two sites about three cm apart in a volume of 0.25 mL per injection site. Immediately after injection an *in vivo* electroporation procedure was applied at the site of injections, using an electroporation device (Cliniporator, IGEA) with linear /hexagonal needle electrodes. The space between the needles was approximately 2 cm and the electroporator was set to 100V. To maintain an average amperage of 0.6 A, a current of 50 V/cm was used. Eight pulses of 20 milliseconds with a 200 millisecond interval between the pulses were applied.

### Blood sampling

Heparinized blood from all 18 animals was collected the day before the BCG vaccination (Day -1), the day before the first (Day 20) and second (Day 41) DNA boost and three weeks after the second DNA boost (Day 63). An extra blood sample was collected, for group 3 animals only, at day 84 for Ag85A T cell epitope mapping. A final blood sample was collected 11 weeks after the second boost (Day 118) and the following day animals were were anesthetized and exsanguinated, and a general pathological examination was performed. Popliteal lymph nodes were harvested after sacrifice, homogenized and cells were kept frozen in DMSO at -80°C for SLA typing. At each time point, heparinized blood was transported within 8 hours after sampling by express courier from Lelystad to the analyzing laboratory in Brussels and cultures were set up the next day.

### Ag85A-specific antibody production

Ag85A-specific porcine IgG was measured by ELISA on 1:200 diluted plasma, using recombinant *E*. *coli* derived histidine-tagged *M*. *tuberculosis* Ag85A [31] for coating (5 μg/mL) and peroxidase-conjugated rabbit anti-pig total IgG (diluted 1:1000) for detection (Sigma-Aldrich, St. Louis, USA). Antibody isotypes were analysed on plasma diluted 1:50, using peroxidase-conjugated mAb-anti-swIgG1, clone 23.49.1 and mAb-anti SwIgG2, clone 341.1.a (Central Veterinary Institute, 1:1000 dilution).

### Lymphoproliferation assays

Lymphoproliferative responses were tested using heparinized whole blood diluted 1:10 in RPMI-1640 medium (Gibco, LifeTech) supplemented with Penicillin/Streptomycin and 2-mercapto-ethanol (5x10^-5^ M) as previously described in human studies [32]. A volume of 180 μl of cells was added to 20μl of the respective antigens (at tenfold final concentration) in round-bottomed 96 microwell plates. Cells were stimulated *in vitro* with bovine PPD (*M*. *bovis* strain AN5, produced at former Pasteur Institute of Brussels, 5 μg/mL final), recombinant *E*. *coli* derived Ag85A of *M*. *tuberculosis* (5 μg/mL final),recombinant *E*. *coli* derived Ag85A of *M*. *avium* subsp *paratuberculosis* [33] (5 μg/mL final), synthetic 20-mer peptides spanning the mature 294 aa Ag85A protein [34] (Innogenetics, Ghent, Belgium 10μg/mL final) or polyclonal mitogen PWM (Lectin from *Phytolacca americana*, Sigma, 20 μg/mL final concentration). Cells incubated in a humidified CO_2_ incubator at 37°C. On day 6, ^3^H-TdR (0,4 μCi/well) was added and the cells were harvested after 20h, using a Skatron Cell Harvester. Filtermats were counted in a BetaPlate LKB scintillation counter. Tests were performed in quintuplicate and results expressed in cpm. Values in non-stimulated cell cultures ranged between 100 and 500 cpm.

### Cytokine assays

For cytokine assays, whole blood was centrifuged at 1,500 rpm for 10 minutes. Plasma was recovered for antibody assays and replaced by a same volume of RMPI-1640 medium supplemented with 10% FCS, antibiotics and 2-mercapto-ethanol (5x10^-5^ M). Cells were adjusted to a concentration of 10^6^ leucocytes/mL in complete RPMI-1640-10% FCS medium and incubated for 7 days in the absence or presence of the different antigens, peptides and mitogen as used in the proliferation assays. Supernatants from three microwells were pooled for each stimulus and stored at -20°C until use. Porcine IFN-γ was quantified by ELISA using purified mouse anti-pig IFN-γ antibody P2G10 for coating and biotinylated detection antibody P2C11 (both BD Pharmingen, San Diego, CA). Sensitivity of ELISA was 5 pg/mL. Porcine IL-6 and IL-10 levels were measured by Luminex Magpix technology, using the Milliplex porcine cytokine test pCYTMAG-23K. This multiplex assay detects IL-6 and IL-10 in a range between 20 pg/mL and 100 ng/mL.

### Intracellular IFN-γ measurement

Intracellular IFN-γ measurement using flow cytometry was performed on fresh isolated PBMCs at day 118. Therefore, PBMCs were isolated from heparinized blood samples by the use of Leucosep tubes, Greiner Bio-One, according to the manufacturer’s instruction. PBMCs (1x10^6^ cells/mL) were cultured in 96-well tissue culture plates in RPMI 1640 medium (Gibco) supplemented with penicillin and streptomycin, 2 mM L-glutamine, 5 x10^-5^ M 2-mercapto-ethanol, 10% FCS, and 5 μg/mL of rAg85A. Cells were maintained in 5% CO_2_ at 37°C. After 3 days of culture, IFN-γ production was analyzed by intracellular staining and fluorescence-activated cell sorter analysis. Brefeldin A (Sigma) was added to the cells at a concentration of 10 μg/mL during the last 4 h of culture and then cells were harvested and washed once in staining buffer. Cell suspensions were transferred to microtiter plates (100 μl per well) and centrifuged for 3 min at 350 x g. Cells were incubated with mAbs directed to porcine CD4 (mouse anti-porcine-CD4: clone 74-12-4, IgG_2b_) and CD8 (mouse anti-porcine-CD8b: clone SL2 (11/295/33), IgG_2a_) for identification of T-cell subpopulations for 30 min at 4°C. The primary antibodies were detected by APC- or FITC-conjugated anti-mouse isotype specific immunoglobulins for CD8^+^ and CD4^+^ T cells respectively (Southern Biotechnology Associates). Next, cells were washed twice in staining buffer and a fixation/permeabilization kit (BD Pharmingen, Cat. nr 555028) was applied according to manufacturer’s instructions. Cells were incubated with phycoerythrin conjugated mouse-anti-pig IFN-γ antibodies (BD Pharmingen, Cat nr. 559812, clone P2G10) diluted in BD perm/wash buffer solution for 30 min at 4°C in the dark followed by two washing steps.

Flow cytometry analyses were performed with a CyAn ADP flow cytometer using Summit Software (both Beckman Coulter). For each sample, at least 1.5 x 10^4^ cells were counted and lymphocytes were gated on the basis of their characteristic forward- and side-scatter profiles. Within the lymphocyte gate CD4^+^, CD8^+^ and IFN γ^+^ cells were analyzed.

### SLA allele typing

Genomic DNA was isolated from frozen popliteal lymph node cells using QIAamp DNA mini kit (Qiagen, cat.no. 51304) and purified by ethanol precipitation. Low resolution MHC class I typing was performed as previously described [35]. In brief, 48 PCR reactions were set up with different primer sets. Each reaction of 10 μl contained 1 μl purified DNA (50–400 ng/μl), 1xPCR Gold buffer (Applied Biosystems, Part no. 4311816), 0.2 mM of each dNTP (Qiagen, cat.no. 201900), 2 mM MgCl_2_, 1 μM forward primer, 1 μM reverse primer, 1xLoading buffer (71 μg/mL cresol red (Sigma-Aldrich, cat.no. 114480), sucrose (15 mg/mL)), 50 μg/mL bovine serum albumin (BSA), 0.06 μl AmpliTaq Gold DNA Polymerase (Applied Biosystems, Part no. 4311816). Co-amplification of the porcine alpha-actin gene was used as the positive control in each reaction. Sequences for the specific typing primers have been described elsewhere [36]. PCR products were analysed on 2% agarose gels (Invitrogen E-Gel, cat.no. G6018-02) to verify the presence of correctly sized amplicons (compared with a molecular marker (50 bp DNA Ladder, Invitrogen, cat.no. 10416–014).

### Candidate peptide selection and SLA peptide affinity analysis

Sequences of Ag85A (Rv3804c) and Ag85B (Rv1886c) were analyzed *in silico* for their potential to be bound by the SLA-1*0401 molecule using the online available peptide predictor *NetMHCpan* v.2.8 (http://www.cbs.dtu.dk/services/NetMHCpan-2.8/). Peptides predicted to be bound were further confirmed as potential candidates by the individual amino acid sequences satisfying requirements for binding in positions 2, 3 and 9 of the SLA-1*0401 binding groove. This was performed using a previously published complete nonamer matrix mapping the SLA-1*0401 peptide binding preferences [37]. Recombinant SLA-1*0401 and beta-2-microglobulin (β_2_m) proteins were produced as previously described [37]. Peptides were tested for their ability to form complexes with the SLA-1*0401 molecule using an immunosorbent assay [38] [39]. A pre-folded, biotinylated FLPSDYFPSV/HLA-A*02:01 [40] complex was used as a standard to convert OD_450_ values to the amount of complex formed using the second order polynomial (Y = a + bX + cX^2^) hereby enabling a direct conversion of the actual peptide concentration offered to the actual concentration of correctly formed pMHC complex. Because the effective concentration of MHC (2–5 nM) used in these assays is below the equilibrium dissociation constant (K_D_) of most high-affinity peptide–MHC interactions, the peptide concentration, ED_50_, leading to half-saturation of the MHC is a reasonable approximation of the affinity of the interaction.

### Statistics

Statistical significance was calculated using one-way ANOVA and Tukey’s post-test (Prism GraphPad software version 5); * : p<0.05; BCG/p85A VS BCG and VS BCG/control pDNA).

## Results

### BCG-pAg85A combination induces strong mycobacteria-specific lymphoproliferative memory responses

**Fig 1:**
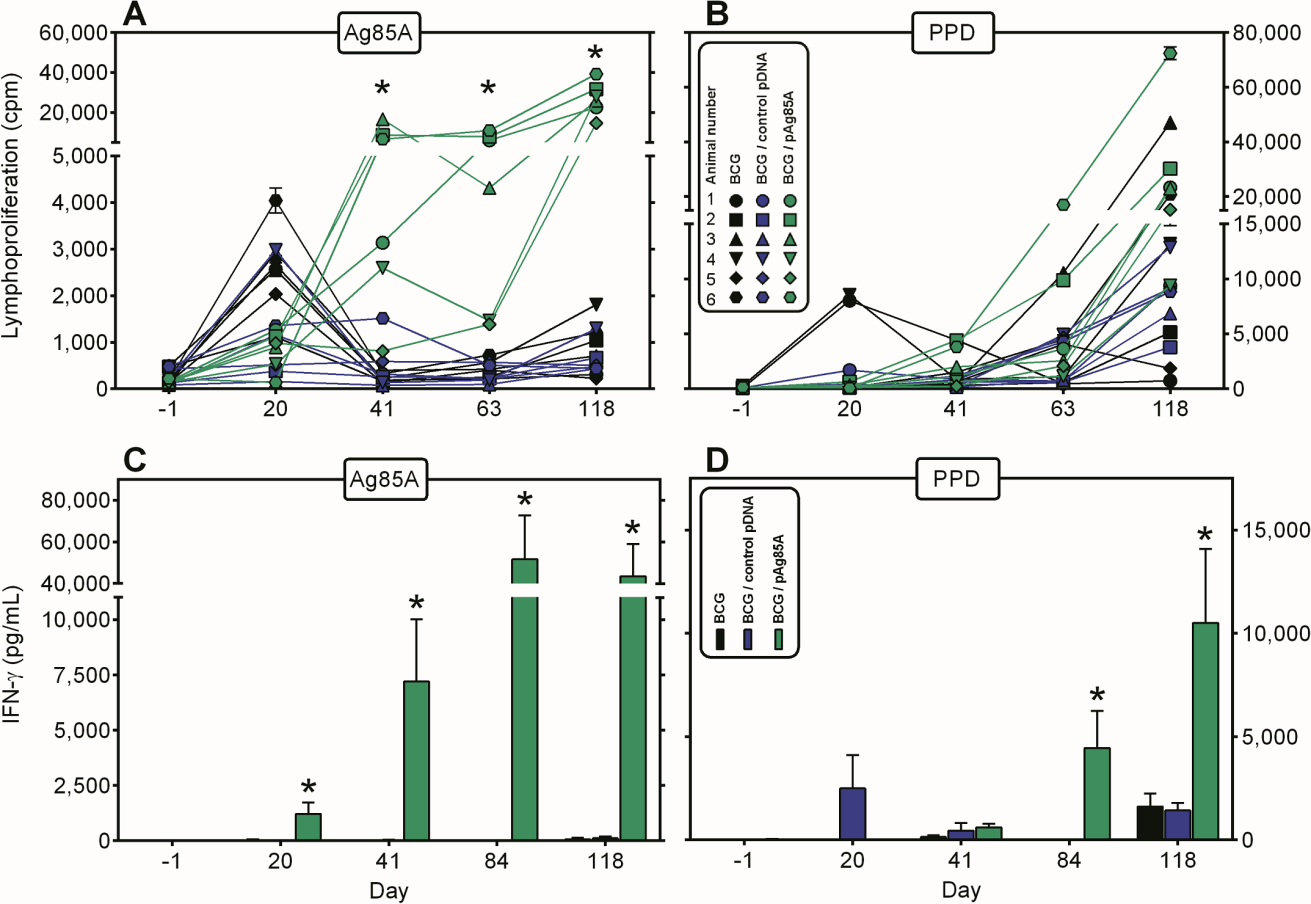
BCG-pAg85A combination induces strong mycobacteria-specific proliferative and IFN-γ responses. Evolution of Ag85A (Fig 1A) and PPD (Fig 1B) specific proliferation throughout the vaccination experiment in group 1 animals vaccinated with only BCG (black symbols), group 2 animals vaccinated with BCG-control vector (blue symbols) and group 3 animals vaccinated with BCG-pAg85A (green symbols). Each point represents mean cpm values of quintuplicate cultures of individual animals. Cells were stimulated *in vitro* with bovine PPD (*M*. *bovis* strain AN5, ex-Pasteur Institute of Brussels, 5 μg/mL final) or recombinant *E*. *coli* derived Ag85A (5 μg/mL final). Evolution of Ag85A (Fig 1C) and PPD (Fig 1D) specific IFN-γ production throughout the vaccination experiment. Bars represent mean IFN- γ levels ± SEM of six animals (pg/mL).

Ag85A-specific lymphoproliferative responses were analysed at day -1, day 20, day 41, day 63 and day 118 in diluted whole blood cultures stimulated for 7 days with recombinant protein (**Fig 1A**). All eighteen animals were tested individually at each time point. Weak proliferative responses were observed on day 20 after BCG priming in animals from all three groups, probably a reflection of the early stimulation of γδ T cells as reported by Lee et al[28]. On day 41, 5/6 animals of group 3 that had received a first pAg85A DNA boost, showed a positive Ag85A-specific proliferative response. On day 63, three weeks after the second pDNA boost, Ag85A specific responses were now detected in 6/6 animals of group 3, albeit that values for two pigs were still lower than for the other four animals. The combined BCG-pAg85A DNA protocol induced a specific memory response, as indicated by a further increase in Ag85A specific responses in all 6 animals of group 3 on day 118, eleven weeks after the second DNA boost. In contrast, Ag85A specific T cell responses decreased to almost baseline values after day 20 in animals of group 1 and 2.

Co-immunization with pDNA encoding Ag85A did not only increase Ag85A-specific responses, but also responses to bovine PPD, the purified protein derivative of tuberculin, which is a mix of at least 200 different proteins of virulent *M*. *bovis* and among which Ag85A is only a minor component (**Fig 1B**). BCG vaccination induced a transient response to PPD in two animals of group 1 at day 20. At day 41, PPD specific responses of about 5,000 cpm were detected in one animal of group 1 and in two animals of group 3. At day 63, PPD specific responses further increased in these latter two pigs. Moreover, one animal of group 1 also scored positive for PPD. Finally at the last time point, day 118, most of the animals showed some proliferative responses to PPD-as expected because they had all been vaccinated with *M*. *bovis* BCG- but responses were clearly higher in 5/6 animals of group 3. One animal of group 1 also showed strong proliferative responses to PPD at this last time point. In contrast to Ag85A specific proliferative responses for which an increase was observed as early as day 41 (after the first DNA boost), the increase in PPD specific response in group 3 animals was only detected in 2/6 animals at day 63, and particularly the memory response at day 118 was dramatically increased.

### BCG-pAg85A combination promotes strong mycobacteria-specific IFN-γ responses

In parallel with the proliferative assays, we measured the IFN-γ levels in 7 day culture supernatants of the diluted whole blood cultures. In order to avoid interference with circulating IFN-γ, cells were washed prior to culture and plasma replaced by RPMI-1640 medium supplemented with 10% FCS. As early as day 20, Ag85A specific IFN-γ responses could be detected in 4/6 animals of group 3 which had received the mix of BCG and pAg85A intradermally, whereas responses in all the other animals were very low or below detection level (**Fig 1C, S1 Table**). Ag85A-specific IFN-γ levels were further boosted by the two additional plasmid DNA injections and at day 118, 5/6 animals had IFN titers that ranged between 18,000 and 103,000 pg/mL. Animal 4 of group 3 showed only a modest Ag85A specific IFN-γ response, reflecting its low lymphoproliferative responses. Ag85A specific IFN-γ titers remained low in all group 1 and group 2 animals.

Modest PPD-specific IFN-γ responses could be detected in 4/6 group 2 animals at day 20 and at day 41, all but one animal in group 1 and group 2 showed some response to PPD. At day 118, all animals of group 1 and 2 produced IFN-γ levels in the range of 1000 to 3000 pg/mL, whereas 5/6 animals of group 3 showed much higher PPD responses, ranging from 6000 to 26000 pg/mL (**Fig 1D, S1 Table**).

### BCG-pAg85A combination induces Ag85A-specific antibodies

**Fig 2:**
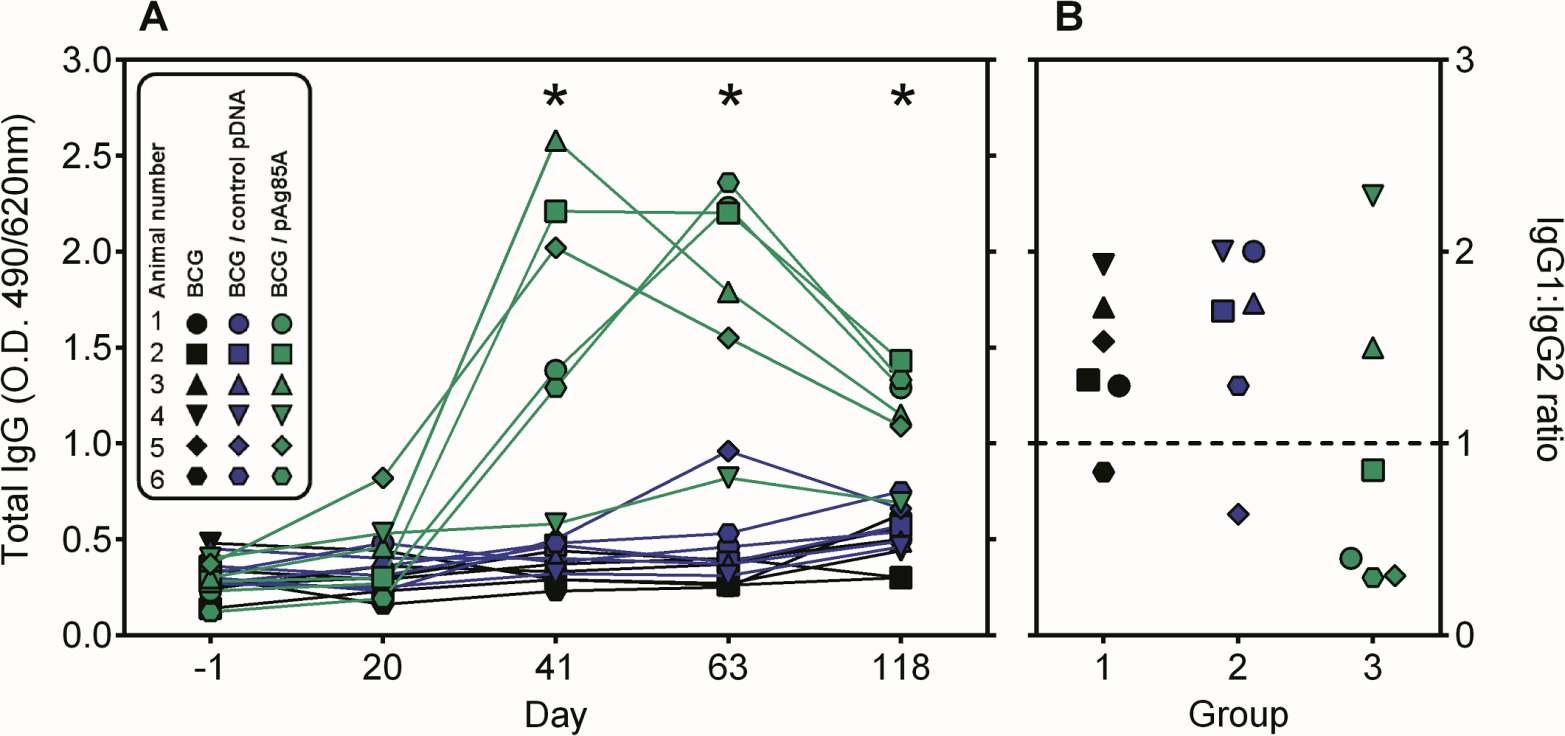
BCG-pAg85A combination induces Ag85A-specific IgG antibodies. **A**. Ag85A-specific antibodies as detected at day -1 before BCG priming and at days 20, 41, 63 and 118 of the vaccination protocol. Mean O.D. values (490/620nm) of individual sera diluted 1:200 are shown for group 1 (black symbols), group 2 (blue symbols) and group 3 (green symbols). **B**. IgG1:IgG2 ratio of Ag85A specific antibodies measured at day 63 of vaccination on sera diluted 1:50.

Ag85A specific IgG antibodies were measured in plasma of the 18 animals. As shown in **Fig 2A** IgG titers were very low and comparable for the three groups prior to vaccination on day -1. BCG vaccination or BCG vaccination combined with empty vector induced a very weak Ag85A specific antibody response, although mean IgG levels on day 118 were twofold higher than on day -1 in two animals of group 1 and group 2. In contrast, Ag85A-specific responses increased in 5/6 animals of group 3 after the first pAg85A boost and antibody titres increased further after a second DNA boost (day 63). On day 118, antibody titres had somewhat declined, but they were still 3 to 10 fold higher than at day -1. Antibody isotypes reflected to some extent the Th1 bias of the induced response as IgG1/IgG2 ratio was inferior to 1 in 4/6 animals of group 3 (**Fig 2B**). However IgG1/IgG2 ratios higher than 1 were measured in animals 3 and 4 (with the lowest overall immune response). Antibodies of group 1 and 2 animals were overall low, but essentially of IgG1 isotype.

### BCG-pAg85A combination induces an IFN-γ producing CD8^+^ T cell compartment

**Fig 3:**
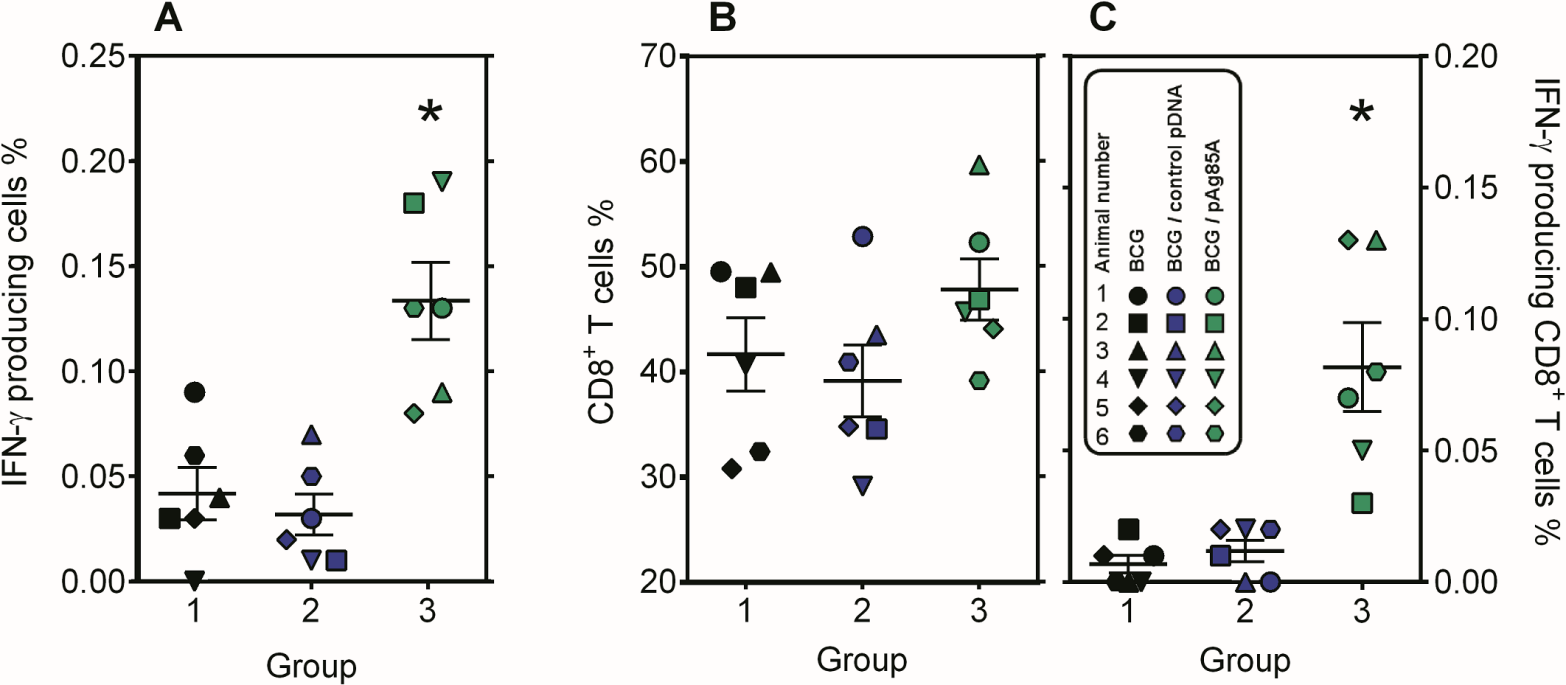
BCG-pAg85A combination induces an IFN-γ producing CD8^+^ T cell compartment. **A**: Intracellular IFN-γ staining of Ficoll purified PBMC cells following 3 day culture of purified PBMC from group 1 (black symbols), group 2 (blue symbols) and group 3 (green symbols) animals, cultured in the presence of rAg85A. B: Percentage of total CD8^**+**^ and **C**: IFN-γ producing CD8^**+**^ T cells as detected by intracellular cytokine staining following 3 days of culture in presence of rAg85A.

In order to find out whether the BCG-pAg85A combination had induced IFN-γ producing Ag85A specific CD8+ T cells, intracellular IFN-γ staining was performed at day 118 on Ficoll purified PBMC from all 18 animals. Cells were stimulated for 3 days with Ag85A protein and the percentage of CD4+ and CD8+ T cells producing IFN-γ was measured. Confirming the whole blood lymphoproliferative and IFN-γ responses, group 3 animals had a higher percentage of IFN-γ producing cells following Ag85A stimulation, than animals of group 1 or 2 (**Fig 3A**). Total % of gated CD8^+^ T cells ranged between 40% and 50% (**Fig 3B).** Somewhat unexpectedly, intracellular IFN-γ staining showed that these CD8^+^ T cells rather than CD4^+^ T cells were the main IFN-γ producers in this experimental setup (**Fig 3C**, data not shown for CD4+ T cells).

### BCG-pAg85A combination increases mycobacteria-specific IL-6 and IL-10 responses

PBMC from pigs vaccinated with the BCG-pAg85A combination also produced higher levels of IL-6 and IL-10 when stimulated with Ag85A and PPD than PBMC of pigs vaccinated with BCG only or with BCG combined with empty vector **(S1 Fig**). IL-6 levels in culture supernatant of cells stimulated with the T cell dependent B cell mitogen PWM were low and comparable for the three groups. IL-10 levels in PWM stimulated culture supernatant were lower in group 2 and group 3 than in group 1, but the difference was not statistically significant.

### Ag85A T cell epitope mapping in vaccinated pigs

**Fig 4:**
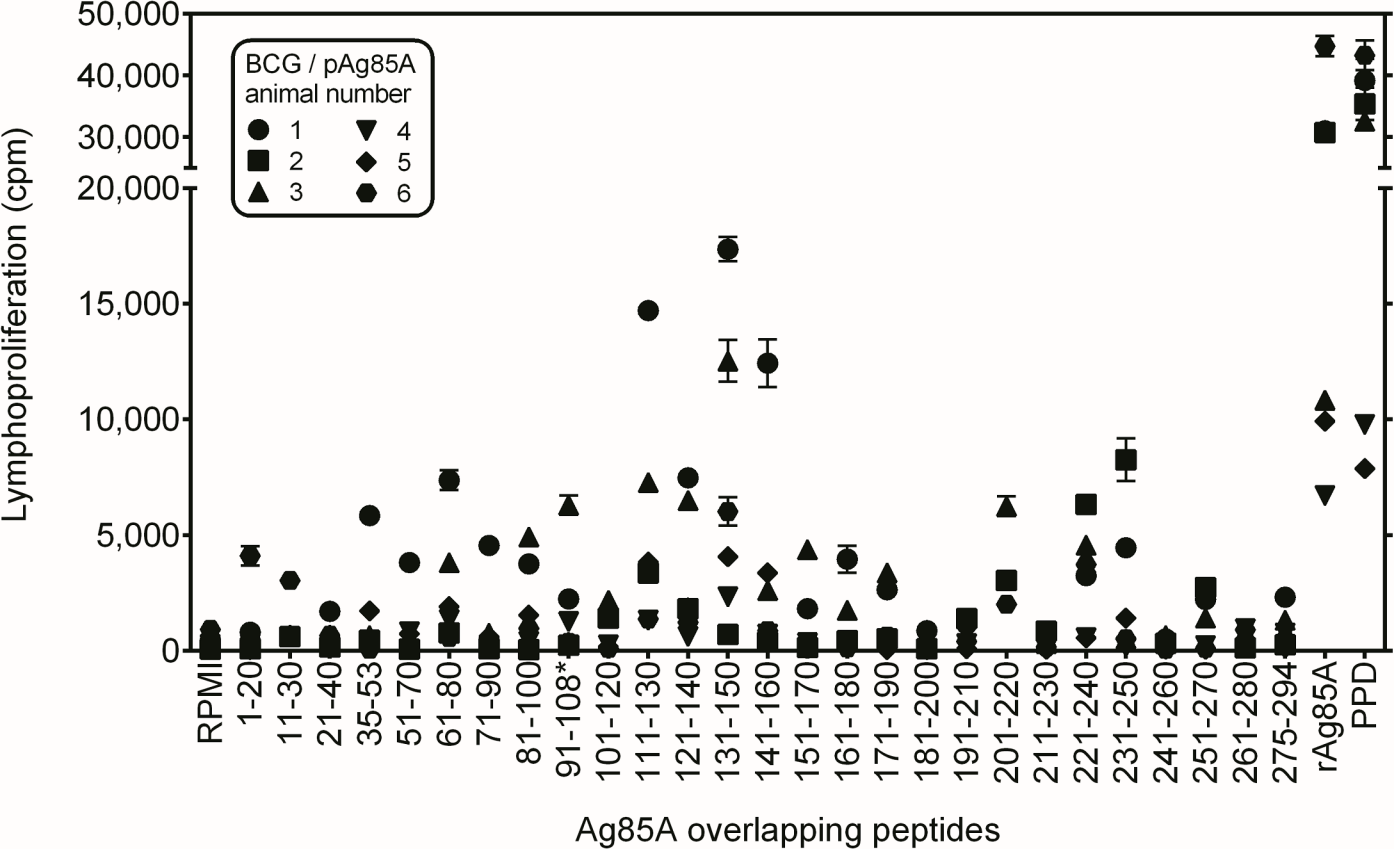
Ag85A T cell epitope mapping in group 3 pigs vaccinated with BCG-pAg85A combination. Ag85A T cell epitope mapping in group 3 pigs vaccinated with BCG-pAg85A combination, as tested on day 84, six weeks after the second pAg85A boost. Each point represents mean cpm values ± SEM of quintuplicate cultures stimulated with the overlapping synthetic 20-mer peptides spanning the mature 294 aa Ag85A protein [34] (Innogenetics, Ghent, Belgium 10μg/mL final), bovine PPD (5μg/mL) or recombinant *Mtb* Ag85A protein (5μg/mL). Different symbols were used to identify the 6 animals. For technical reasons, peptide 10 spanning aa 91–110 of Ag85A was replaced by the corresponding peptide of Ag85B spanning aa* 91–108. This sequence is identical to the Ag85A sequence except for a Gly107Gln shift.

We have previously shown in experimental mouse models, that plasmid DNA vaccination is a very powerful tool for the broadening the immune repertoire and the identification of murine CD4^+^ and CD8^+^ T cell epitopes of a protein antigen, using synthetic 20-mer peptides for initial screening [41–43]. Because of the strong Ag85A specific responses observed on day 63 in group 3 animals, an additional blood sample was collected for these 6 pigs on day 84, 6 weeks after the second DNA boost. Cells were stimulated with synthetic peptides spanning the entire sequence of mature Ag85A protein [44]. Proliferative and IFN-γ responses were analysed in 7 day cultures. As shown in **Fig 4**, a number of peptides induced strong proliferative responses by T cells of group 3 pigs. Interestingly, stimulation with peptide 14 (Ag85A_131-150_) stimulated a positive proliferative response in 5/6 animals, suggestive of the presence of a promiscuously recognized T cell epitope. Other peptides such as Ag85A_61-80_ and Ag85A region spanning aa 221–250 also induced proliferative responses, particularly in animals 1 and 2. IFN-γ levels measured in response to peptide stimulation closely reflected the proliferative responses. Significant IFN-γ levels were detected in cultures stimulated with Ag85A_131-150_ in all six group 3 animals (**Table 2**).

**Table 2:** T cell epitope mapping in group 3 animals vaccinated with the BCG-pAg85A combination.

Overlapping peptide (aa)	BCG / pAg85A animal number					
	1	2	3	4	5	6
RPMI	<5 ^a^	64	<5	<5	132	1,620
1–20	<5	305	<5	<5	84	**13,875**
11–30	<5	1,115	<5	<5	80	<5
21–40	<5	681	<5	128	142	<5
35–53	4,594	438	74	<5	2,105	<5
51–70	5,083	<5	<5	<5	3,125	<5
61–80	8,179	354	579	75	3,611	444
71–90	217	<5	<5	<5	289	<5
81–100	1,900	56	2,968	170	1,547	<5
91–108*	<5	2,332	2,377	308	748	<5
101–120	150	1,646	1,177	<5	319	<5
111–130	15,124	2,879	1,415	33	961	<5
121–140	16,699	2,367	306	<5	1,023	<5
131–150	**11,114**	**1,754**	**5,306**	**868**	**11,072**	**4,687**
141–160	9,718	232	1,330	**894**	3,006	837
151–170	1,020	<5	257	71	349	<5
161–180	1,501	<5	556	37	463	<5
171–190	328	<5	1,066	22	170	<5
181–200	33	<5	8	17	<5	<5
191–210	266	<5	156	38	560	<5
201–220	21	<5	1,315	<5	<5	197
211–230	46	<5	13	<5	<5	218
221–240	470	4,455	1,075	<5	303	2,980
231–250	**11,639**	**13,693**	423	<5	1,018	665
241–260	40	<5	<5	<5	<5	66
251–270	410	317	<5	11	161	163
261–280	31	<5	<5	157	728	1,240
275–294	57	<5	<5	<5	114	77
PPD _bov_	5,467	12,622	2,108	20	3,143	3,345
Ag85A _tub_	145,331	50,084	68,850	3,712	27,544	14,506

a: IFN-γ levels (expressed in pg/mL) in 7 day culture supernatants of whole blood cells (10^6^ leucocytes/mL) of the six pigs of group 3, collected at day 84, six weeks after the second DNA boost. Cells were stimulated with synthetic overlapping 20-mer peptides of Ag85A (10μg/ mL). Reponses against immunodominant peptide14 and peptide 24 are highlighted in bold.

*: 18-mer peptide spanning the sequence of Ag85B.

**Fig 5:**
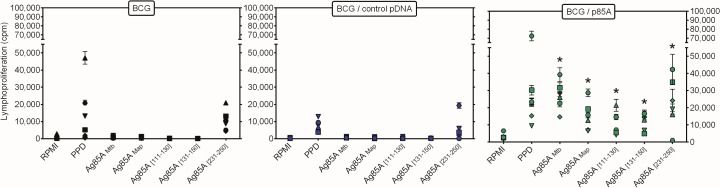
Ag85A T cell epitope mapping in vaccinated pigs. Lymphoproliferative responses at day 118 in animals vaccinated with BCG (left Fig), with BCG combined with empty vector (middle Fig) or BCG combined with pAg85A (right Fig) of diluted whole blood cultures stimulated for 7 days with medium (T), bovine PPD, recombinant Ag85A of *M*.*tuberculosis*, recombinant Ag85A of *M*. *avium* subsp. *paratuberculosis* (*Map*), 85A _111–130_, 85A _131–150_ or 85A _231–250_. Each point represents mean cpm values of quintuplicate cultures of individual animals.

In a final experiment, the proliferative response of all eighteen animals was tested at day 118 in response to Ag85A _131–150_ and to two Ag85A peptides Ag85A _111–130_ and Ag85A _231–250_ (that scored negative in the MHC predictions, but were recognized by some group 3 animals). As shown in **Fig 5**, positive responses were detected to bovine PPD and Ag85A _231–250_ in animals of all three groups, although magnitude of the response was lower in animals that had been vaccinated with BCG alone or BCG combined with empty vector. Responses to Ag85A_111-130_ and Ag85A_131-150_ were only measured in group 3 animals and this was also the case for responses against the recombinant Ag85A of *M*. *tuberculosis*. Cross-reactive responses against recombinant Ag85A of *M*. *avium* subsp. *paratuberculosis (Map)* were only detected in group 3.

### SLA allele typing, candidate peptide selection and SLA peptide affinity analysis

Using the online available prediction algorithm *NetMHCpan* (http://www.cbs.dtu.dk/services/NetMHCpan-2.8/), which has been proven as a solid tool in successful peptide predictions beyond humans [45–47], we analysed the sequence of Ag85A for possible SLA class I binding motifs. Ag85A_131-150_ turned out to encompass a predicted strong binding epitope for SLA-1*0401 and SLA-1*0801 alleles. Typing of group 3 animals for SLA-1 alleles expression identified the following alleles: animal 1: 08XX, 11XX; animal 2: 04XX, 11XX; animal 3: 08XX, 13ms21; animal 4: 01XX, 11XX; animal 5: 08XX, 11XX; animal 6: 04XX, 15XX [36,48]. Hence three animals expressed the SLA-1*08XX, two animals expressed the SLA-1*04XX allele whereas one animal (number 4) expressed the SLA-1*01XX allele. This latter animal was overall the lowest responder in lymphoproliferation, IFN-γ secretion and antibody response.

One of the most commonly occurring SLA alleles, the SLA-1*0401 [49], has recently been mapped for its peptide binding preferences [37]. Nine Ag85A and Ag85B derived peptides were selected based on *in silico NetMHCpan* predictions with rank scores of <2.0 (**Table 3**). The peptides were all present as part of the longer Ag85A_131-150_ peptide. A single peptide having a *NetMHCpan* rank score of 7.00/4.00 was included because of a specific SLA-1*0401 binding groove P9 pocket match [37]. All nine candidate peptide epitopes were analysed *in vitro* for their specific binding affinities by the SLA-1*0401 molecule. *NetMHCpan* rank scores and actual binding affinities for each of the candidate peptide epitopes are shown in **Table 3**.

**Table 3:** SLA peptide affinity analysis.

Peptide sequence	Ag85	Amino acids	NetMHCpan Rank score^a^	SLA-1*0401
			(SLA-1*0401/SLA-1*0801)	affinity (nM)^b^
ASSALTLAI	A	129–137	1.50/3.00	10
**SSALTLAIY**	A	130–138	0.40/0.50	**17**
LAIYHPQQF	A	135–143	7.00/4.00	20,000
ASSALTLAIY	A	129–138	0.25/0.40	1,971
**AIYHPQQFVY**	A	136–145	0.50/0.12	**5**
LSMAGSSAM	B	125–133	0.80/0.40	375
**SSAMILAAY**	B	130–138	0.20/0.17	**106**
GSSAMILAAY	B	129–138	0.40/0.30	7,755
ILAAYHPQQF	B	134–143	1.50/0.50	6,043

a: Peptides having *NetMHCpan* rank scores of <2.00 were considered as predicted for high affinity binding by the respective SLA molecules of interest (SLA-1*0401 and SLA-1*0801).

b: Binding affinity of the 9 synthetic peptides determined for SLA-1*0401 as described by Sylvester-Hvid *et al* [38]. Peptides with strong binding affinity are indicated in bold.

The tested peptides were bound to the SLA-1*0401 molecule with affinities in a range of 5–20,000 nM. One very strong SLA-1*0401 binding peptide spanning aa 136–145 was identified with an affinity of 5 nM for SLA-1*0401. Furthermore, this peptide had a rank score 0.5 for the SLA-1*0401 binding prediction, and an even better rank score of 0.12 for SLA-1*0801. Ag85B _134–143_ showed only a low binding affinity of 6,043 nM for SLA-1*0401, indicating that the predicted CD8^+^ epitope spanning aa 136–145 is probably specific for the Ag85A member of the Ag85 complex. Ag85A _130–138_ was also identified as a strong SLA-1*0401 binder with an affinity of 17 nM. The promiscuously recognized peptide 14 (Ag85A_131-150_) does not have the serine in position 130, but this serine 130 is present in peptide 13 (aa 121–140), recognised by animals 1 and 3. The synthetic peptide spanning the corresponding Ag85B_130-138_ sequence also bound with strong affinity to SLA-1*0401 molecule, suggesting that besides an Ag85A specific CD8^+^ epitope (spanning aa 136–145), there may be a cross-reactive epitope (spanning aa 130–138) shared between Ag85A and Ag85B in this particular region.

## Discussion

Designing vaccines that mimic virulent *M*. *tuberculosis* or *M*. *bovis*, promoting phagolysosomal translocation into the cytosol is likely critical to enhance CD8^+^ T cell activation [50]. DNA vaccines stimulate both the exogenous (MHC class II restricted) and the endogenous (MHC class I restricted) antigen presentation pathways, and so far DNA vaccines are the only type of *genuine* subunit vaccines, expressing-in contrast to viral vectors or recombinant BCGs secreting listeriolysin/ perfringolysin [50]- only the transgene, with a capacity to induce strong CD8^+^ T cells. DNA vaccines are particularly suited for homologous prime/boost strategies, devoid of any concerns about anti-vector immunity.

Using a swine codon optimized plasmid and improved delivery by *in vivo* electroporation, we were able to improve dramatically the immunogenic potential of BCG. Our study confirms the well-known potential of *in vivo* electroporation for increasing immunogenicity of DNA vaccines in larger mammalian species, such as cattle, goats, sheep, swine and non-human primates [51] [52] [53]. To our knowledge, DNA vaccines encoding mycobacterial antigens have not been studied in the porcine model, although pigs are an ideal species for vaccine research [25]. As compared to cattle, they are relatively cheap and because of their size as adults (which is comparable to that of humans), they can be housed in large groups. Furthermore, extensive information on swine leukocyte antigens is available and it is possible to predict cytotoxic T cell epitopes. Like humans, swine are a natural host to *Mycobacterium* species [54] and wild boar and free-range pigs can be infected by *Mycobacterium tuberculosis* complex and contribute to the spread of bovine tuberculosis [55]. Swine develop similar pathological lesions to those seen in humans following *M*.*bovis* infection [56] and by providing a local pulmonary structure similar to that in humans, the (mini)pig model highlights aspects that could be key to a better understanding of particularly latent tuberculosis infection in humans [57].

Here we have shown that co-administration of a DNA vaccine encoding the protective antigen Ag85A of *M*. *tuberculosis* with the existing *M*. *bovis* BCG vaccine, can induce a long-lived mycobacteria-specific IFN-γ producing Th1 cell and B cell response in *Sus scrofa domestica*. Immune responses in pigs co-vaccinated with BCG and empty vector were much weaker and comparable to responses in pigs vaccinated with non-formulated BCG. Intracellular cytokine staining showed that IFN-γ producing Ag85A specific CD8^+^ T cells were induced by the BCG-pAg85A combination. Using synthetic peptides spanning the entire mature Ag85A sequence, a promiscuous Ag85A_131-150_ specific IFN-γ response was demonstrated in all group 3 animals. This peptide was predicted to contain strong candidates for binding by the SLA-1*0401 and SLA-1*0801 molecules, expressed by 5/6 group 3 animals. These results strongly suggest that the Ag85A_131-150_ specific promiscuous T cell response detected in group 3 animals was indeed mediated by SLA1*0401 and SLA-1*0801 MHC class I restricted CD8^+^ cells, further highlighting the strong potential of DNA vaccines to induce this T cell subset.

Immune responses directed against PPD were induced in all animals by the BCG vaccine, but to a higher extent in pigs vaccinated with the BCG-pAg85A combination. In an experimental mouse model, we have observed that the combination of BCG with plasmid DNA encoding a particular mycobacterial antigen, can increase and broaden the antigenic repertoire of BCG-induced responses and induce T cell reactivity to other mycobacterial antigens [58]. These results suggest that boosting with coding plasmid DNA (but not with empty vector) can induce bystander activation, probably through the creation of a Th1 type cytokine milieu.

Besides an activation of immune responses against non plasmid encoded BCG- expressed antigens caused by the DNA booster injections, we have observed that BCG can exert an adjuvant effect on the plasmid DNA-induced responses. Thus, in an experimental mouse model, we showed that ovalbumin (OVA)-specific IFN-γ titers were higher in BCG/pOVA co-vaccinated mice than in mice only vaccinated with pOVA DNA, although both groups had received the same plasmid dose three times [58]. The BCG cell wall is composed of several pathogen associated molecular patterns (PAMPs) that are able to interact with different pathogen recognition receptors, such as TLR2, TLR4, TLR9, NOD2 and Mincle involved in the induction of innate immune responses. Plasmid DNA vaccines also have intrinsic PAMP properties, because they can activate TLR9 through their bacterial CpG motifs and stimulate TBK1-dependent innate immune signalling pathways through their double-stranded structure [59]. Therefore, it is tempting to speculate that there might be synergies on TLR-induced innate immune responses provided by simultaneous BCG and pDNA administration, knowing, for example, that TLR9 regulates Th1 responses and cooperates with TLR2 in mediating optimal resistance to *Mtb* [60]. Moreover, TLR9 signalling seems to be critical for the induction of effective CD8^+^ T-cell responses through cross-priming following the initial pDNA immunization [61]. In contrast to mice, TLR9 expression is mainly limited to plasmacytoid dendritic cells in pigs (as it is in humans). Therefore, the increased immune potential of BCG we have obtained here in pigs is an indirect indication that the combined BCG-pDNA approach will also work in humans. It is tempting to speculate that the combination protocol could even be used for other bacterial, viral and protozoal human pathogens.

The combination of BCG with DNA vaccines as we have described here, could be used as well to increase immune responses against antigens, which are only poorly immunogenic upon BCG vaccination, such as the so-called latency associated antigens, the expression of which is up regulated in *M*. *tuberculosis* grown in conditions of starvation and dormancy [62] [63].

In summary, our study provides a proof of concept in a large mammalian species for a new TB vaccine based on a BCG-pDNA combination. A number of phase 1 clinical trials have now reported on the safety and immunogenicity of DNA vaccines, targeting mostly viral pathogens [64]. Our findings on the BCG-pDNA combination open interesting prospects for testing this new type of tuberculosis vaccine in pigs and eventually in non-human primates.

## Supporting Information

S1 Fig Increased mycobacteria-specific IL-6 and IL-10 production in pigs vaccinated with the BCG-pAg85A combination. IL-6 and IL-10 content (pg/mL) in culture supernatants of cells from all 18 animals collected at day 118 and tested with Milliplex porcine cytokine kit pCYTMAG-23K, using MAGPIX technology. Results show the mean IL-6 and IL-10 levels detected in group 1 (black bars), group 2 (blue bars) and group 3 (green bars) in non-stimulated cells (RPMI) or cells stimulated with recombinant Ag85A, bovine PPD or the polyclonal pokeweed mitogen PWM (Lectin from *Phytolacca americana*, Sigma, 20 μg/mL final concentration). Results show the mean titres ± SEM values of the six animals/group.
(TIF)

S1 Table IFN-γ levels in 7 day supernatant of leucocyte cultures (whole blood diluted 1:10) stimulated with recAg85A (5 μg/mL) or bovine PPD (5 μg/mL) and tested at different time points of the follow-up. Supernatants from three wells were pooled. Results are reported in pg/mL. ND: not done
(DOCX)
