# Supplementary figures and images for: Increased B and T Cell Responses in M. bovis Bacille Calmette-Guérin Vaccinated Pigs Co-Immunized with Plasmid DNA Encoding a Prototype Tuberculosis Antigen
Source: PLoS One. 2015 Jul 14;10(7):e0132288. doi: 10.1371/journal.pone.0132288 (PMC4501720; doi:10.1371/journal.pone.0132288)

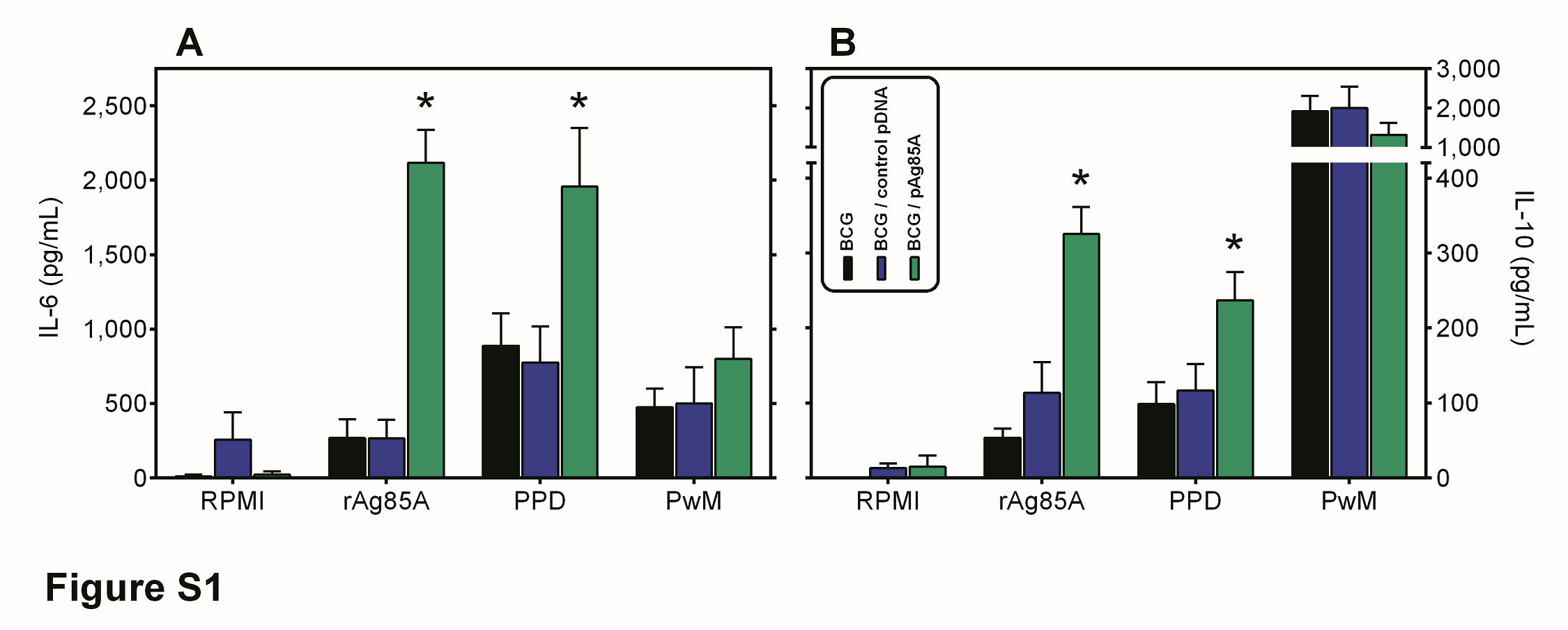

Supplement: S1 Fig — IL-6 and IL-10 content (pg/mL) in culture supernatants of cells from all 18 animals collected at day 118 and tested with Milliplex porcine cytokine kit pCYTMAG-23K, using MAGPIX technology. Results show the mean IL-6 and IL-10 levels detected in group 1 (black bars), group 2 (blue bars) and group 3 (green bars) in non-stimulated cells (RPMI) or cells stimulated with recombinant Ag85A, bovine PPD or the polyclonal pokeweed mitogen PWM (Lectin from Phytolacca americana, Sigma, 20 μg/mL final concentration). Results show the mean titres ± SEM values of the six animals/group. (TIF) [file pone.0132288.s001.tif]
