# Supplementary material for: Increased B and T Cell Responses in M. bovis Bacille Calmette-Guérin Vaccinated Pigs Co-Immunized with Plasmid DNA Encoding a Prototype Tuberculosis Antigen
Source: PLoS One. 2015 Jul 14;10(7):e0132288. doi: 10.1371/journal.pone.0132288 (PMC4501720; doi:10.1371/journal.pone.0132288)
Supplement: S1 Table — Supernatants from three wells were pooled. Results are reported in pg/mL. ND: not done (DOCX) [file pone.0132288.s002.docx]

**Supporting information Table 1**. Evolution of Ag85A and PPD specific IFN-γ levels.

| **Ag85A** | **Animal number** |  |  |  |  |  |
| --- | --- | --- | --- | --- | --- | --- |
|  |  | **d-1** | **d20** | **d41** | **d84** | **d118** |
| BCG | 1 | 19 | 13 | <5 | ND | <5 |
|  | 2 | <5 | <5 | <5 | ND | <5 |
|  | 3 | <5 | 48 | <5 | ND | 10 |
|  | 4 | <5 | <5 | <5 | ND | <5 |
|  | 5 | <5 | 154 | <5 | ND | <5 |
|  | 6 | <5 | <5 | <5 | ND | 393 |
| BCG / control pDNA | 1 | 37 | <5 | <5 | ND | 15 |
|  | 2 | <5 | <5 | 95 | ND | 477 |
|  | 3 | <5 | <5 | <5 | ND | 21 |
|  | 4 | <5 | <5 | <5 | ND | 29 |
|  | 5 | 20 | 20 | 96 | ND | <5 |
|  | 6 | <5 | <5 | <5 | ND | 135 |
| BCG / pAg85A | 1 | <5 | 77 | 14,921 | 145,331 | 103,600 |
|  | 2 | <5 | 28 | 4,008 | 50,084 | 43,237 |
|  | 3 | <5 | 489 | 16,921 | 68,850 | 71,492 |
|  | 4 | <5 | 1,685 | 530 | 3,712 | 3,717 |
|  | 5 | <5 | 3,347 | 3,425 | 27,544 | 18,586 |
|  | 6 | <5 | 1,671 | 3,407 | 14,506 | 20,416 |

| **PPD** | **Animal number** | **d-1** |  |  |  |  |
| --- | --- | --- | --- | --- | --- | --- |
|  |  |  | **d20** | **d41** | **d84** | **d118** |
| BCG | 1 | 54 | 29 | <5 | ND | 46 |
|  | 2 | <5 | <5 | 428 | ND | 1,262 |
|  | 3 | <5 | <5 | 80 | ND | 1,154 |
|  | 4 | 38 | <5 | <5 | ND | 3,524 |
|  | 5 | 26 | 61 | 330 | ND | 284 |
|  | 6 | <5 | <5 | 36 | ND | 3,437 |
| BCG / control pDNA | 1 | 39 | <5 | <5 | ND | 740 |
|  | 2 | <5 | 9,948 | 6 | ND | 226 |
|  | 3 | <5 | 4,095 | 2,268 | ND | 1,638 |
|  | 4 | <5 | 674 | 70 | ND | 1,410 |
|  | 5 | 30 | <5 | 296 | ND | 1,707 |
|  | 6 | 2 | 219 | 43 | ND | 2,800 |
| BCG / pAg85A | 1 | <5 | <5 | 511 | 5,467 | 14,316 |
|  | 2 | <5 | <5 | 1,207 | 12,622 | 26,033 |
|  | 3 | 33 | 9 | 493 | 2,108 | 8,091 |
|  | 4 | 84 | <5 | 68 | 20 | 795 |
|  | 5 | <5 | <5 | 330 | 3,143 | 5,992 |
|  | 6 | 32 | <5 | 988 | 3,345 | 7,785 |
